# Categories of Aortic Stenosis: What’s New and the Clinical Implications

**DOI:** 10.3390/medicina62050819

**Published:** 2026-04-25

**Authors:** Jamie Sin Ying Ho, Gerlyn Zhixuan Wong, Aaron Kwun Hang Ho, Aloysius S. T. Leow, Joy Yi-Shan Ong, William Kong, Swee Chye Quek, Andrew Fu Wah Ho, Ching Hui Sia, Hoai Thi Thu Nguyen, Tiong Cheng Yeo, Kian Keong Poh

**Affiliations:** 1 Department of Medicine, National University Hospital, Singapore 119228, Singaporegerlynwong2002@gmail.com (G.Z.W.); aaronhkh@gmail.com (A.K.H.H.); 2 Department of Cardiology, National University Heart Centre Singapore, Singapore 119228, Singapore; 3 Division of Paediatric Cardiology, Department of Paediatrics, Khoo Teck Puat-National University Children’s Medical Institute, National University Hospital, Singapore 119074, Singapore; 4 Department of Emergency Medicine, Singapore General Hospital, Singapore 168581, Singapore; 5 Prehospital Emergency Research Center, Duke-NUS Medical School, Singapore 169857, Singapore; 6 Vietnam National Heart Institute, Bach Mai Hospital, Hanoi 10000, Vietnam; hoainguyen1973@gmail.com; 7 Department of Internal Medicine, VNU-University of Medicine and Pharmacy, Hanoi 10000, Vietnam

**Keywords:** aortic stenosis, prognosis, aortic valve replacement

## Abstract

Aortic valve stenosis (AS) is assessed by echocardiography in clinical practice. Conventionally, the aortic valve area, peak transaortic valve velocity/gradient and the mean transvalvular gradient determine if the AS is categorized as mild, moderate or severe. Recently, the entity of paradoxical low-flow, low-gradient AS despite normal left ventricular ejection fraction (LVEF) was described and flow (as determined by stroke volume indexed to body surface area) was used to further categorize AS. The new European Society of Cardiology (ESC) and the European Association for Cardio-Thoracic Surgery (EACTS) guidelines in 2025 recommended a new phenotype-based classification, which improved the prognostication of AS. There are now five phenotypes: (1) concordant high-gradient AS; (2) low-flow, low-gradient AS with reduced LVEF; (3) low-flow, low-gradient AS with preserved LVEF; (4) normal-flow, low-gradient AS with preserved LVEF; and (5) discordant high-gradient AS. These appear to have different underlying pathophysiology, and hence prognostication and therapy. In addition, categories of AS in the setting of reduced LVEF are further divided based on their responses to dobutamine or exercise stress, which may result in different therapeutic strategies. In the transaortic valvular replacement (TAVR) versus the surgical aortic valve replacement (SAVR) era, the classification of these AS groups may have differing implications on the appropriate interventions. Furthermore, there are investigations on the effect of AS on the left ventricle and other chambers and stages of AS based on the extent of cardiac damage, which may have important prognostic value post-AVR. On the other spectrum, there are new developments in imaging analysis, such as using artificial intelligence. This state-of-the-art paper will comprehensively review the important updates in AS and its clinical implications.

## 1. Introduction

Aortic stenosis is one of the most common forms of valvular heart disease, with increasing prevalence with age. The pooled prevalence of AS in those aged ≥75 years was 12.4% in a systematic review, with severe disease in 3.4% of the elderly population [1]. Severe AS is associated with poor outcomes, and the mortality rate at 4 years increases from 25% in mild AS to 45% in severe AS without aortic valve replacement [2]. AS is often diagnosed when asymptomatic, on detection of an ejection systolic murmur on physical examination, or incidentally identified in echocardiogram performed for other indications.

Aortic valve replacement (AVR) is the only effective treatment for AS at present, whether transcatheter or surgical, and delayed intervention is associated with adverse cardiac remodeling, particularly left ventricular hypertrophy and left ventricular systolic dysfunction, causing symptoms from heart failure, angina and syncope. In patients with LV systolic dysfunction, reduction in afterload after AVR significantly improves LVEF, and improves survival [3]. However, it is an invasive procedure associated with risk of stroke, endocarditis, bleeding and mortality, hence careful patient selection and appropriate timing of intervention is required.

Based on the current international guidelines, AVR is indicated for patients with severe AS when symptomatic, with reduced LV ejection fraction without another cause, or with high-risk features even if asymptomatic [4]. In the past, invasive measurements via cardiac catheterization were deemed as the gold standard. With the advancements in echocardiography, studies have found good correlation of measurements using Doppler methods and invasive measurements [5]. Catheterization is also associated with adverse events such as stroke, and is invasive, hence the modern gold standard for evaluation of AS is via echocardiogram and cardiac catheterization is not recommended except in cases where echocardiography is non-diagnostic or is discrepant with clinical data [6]. However, up to 40% of cases demonstrate discordant echocardiographic hemodynamic parameters, the main causes of which are measurement inaccuracies and low-flow states [7,8], leading to inaccurate categorization of the severity of AS, potentially causing delays in appropriate intervention.

The new European Society of Cardiology (ESC) and the European Association for Cardio-Thoracic Surgery (EACTS) guidelines in 2025 recommended a new phenotype-based classification, which improved the prognostication of AS [4]. There are now five phenotypes: (1) concordant high-gradient AS; (2) low-flow, low-gradient AS with reduced LVEF; (3) low-flow, low-gradient AS with preserved LVEF; (4) normal-flow, low-gradient AS with preserved LVEF; and (5) discordant high-gradient AS. These appear to have different underlying pathophysiology, and hence prognostication and therapy. In this review, we will discuss the recent updates on the diagnostic classification of AS, their implications on prognostic stratification and evidence-based treatment.

## 2. Materials and Methods

A narrative review is performed to summarize and interpret the current literature on the categorization of aortic stenosis and its clinical implications. A search of the literature on PubMed was performed to identify major themes in the categorization of AS and seminal articles that are relevant to this review from inception to 8 December 2025. The search terms included “aortic stenosis” and “category”. Only articles in English were included. These articles were reviewed by two authors (JSYH and WZG), and data were extracted and summarized in this review.

## 3. Conventional Hemodynamic Classification of AS

For many decades, the severity of AS has been categorized into mild, moderate and severe based on the hemodynamic measurements of peak transaortic jet velocities, mean gradients and aortic valve areas (AVA) (Table 1) [9]. One limitation of this classification is that the transaortic velocities and gradient vary with volume flow rate, and in patients with low volume flow rate, such as those with left ventricular (LV) dysfunction or small LV, severe AS may be missed. Even in patients with normal LV function, there was up to 40% discrepancy in the categorization based on the three parameters, particularly in patients with low stroke volume [10]. Minners et al. showed that nearly one-third of patients were diagnosed with severe AS based on AVA criterion but not based on mean gradient, highlighting the inconsistencies of echocardiographic criteria. Possible reasons for the discordance between AVA and mean gradient in patients despite preserved LVEF may include measurement errors, particularly underestimation of left ventricular outflow tract (LVOT) diameter which can overestimate the severity of AS, small body size with associated lower stroke volume and paradoxical low-flow AS. These limitations prompted the development of other classifications of AS to better grade AS severity for accurate prognostication with implications on treatment.

### 3.1. Phenotype-Based Classification of AS

The flow status significantly impacted the assessment of AS and its prognosis, which led to the modern phenotype-based classification. In 2007, Hachicha et al. described a population of paradoxical low-flow, low transvalvular gradient AS [12], despite more LVEF, who are patients with often under-estimated AS severity and delay in AVR. This significant study identified the pattern of low transvalvular flow and low gradients despite normal LV ejection fraction as a distinct group with more advanced stage of AS and increased mortality.

The 2025 ESC/EACTS guidelines recommended a new phenotype-based classification, which improves prognostication of AS [4]. Flow is determined by stroke volume indexed to body surface area (SVi), with low flow defined as SVi ≤ 35 mL/m^2^ on Doppler echocardiography, a widely accepted cut-off based on the seminal study by Hachicha et al. in 2007 [12]. There are now five recognized phenotypes based on the integration of gradient, flow, and LVEF (Table 2). These phenotypes differ in their underlying pathophysiology and have important implications for diagnosis and management. Although the diagnosis and management of concordant HG AS is straightforward, the diagnosis and management of LF-LG and NF-LG AS remains controversial.

### 3.2. Concordant High-Gradient AS

Patients with concordant high-gradient AS represent the classic severe AS with raised mean gradient, peak velocity (V_max_) and reduced AVA (AVA ≤ 1.0 cm^2^, V_max_ ≥ 4.0 m/s, and mean gradient ≥ 40 mmHg). They unequivocally have severe AS, and there are clear indications for intervention if symptomatic or meeting other criteria such as reduced LVEF < 50% without an alternative cause, or adverse prognostic features such as very high V_max_, elevated natriuretic peptides, severe valve calcification, or rapid V_max_ progression. Recently, four randomized controlled trials compared watchful waiting versus early AVR in patients with asymptomatic high-gradient AS, and a meta-analysis of 1427 patients found that early AVR significantly reduced the incidence of the composite outcome when compared with conservative management, including all-cause mortality, hospitalization for cardiovascular causes, stroke, and myocardial infarction, but not cardiovascular mortality [13]. Patients with low-flow high-gradient AS has particularly worse outcomes, compared to patients with normal-flow HG AS or LG AS [14]. Hence, early intervention should be considered in patients with concordant high-gradient AS, particularly if procedural risk is low.

### 3.3. Low-Gradient AS

**Figure 1:**
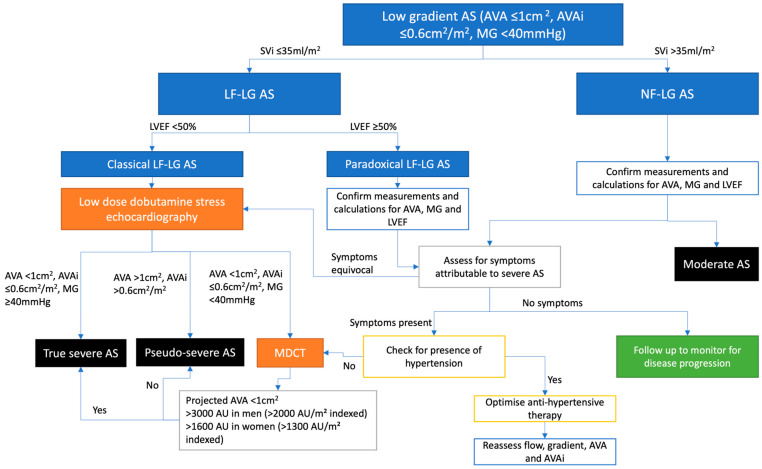
Algorithm for the evaluation of low-gradient aortic stenosis with discordant phenotypes. AS = aortic stenosis; AU = Agatston Units; AVA = aortic valve area; AVAi = aortic valve area indexed to body surface area; LF = low flow; LG = low gradient; LVEF = left ventricular ejection fracture; MDCT = multidetector computed tomography; MG = mean gradient; NF = normal flow.

In contrast to high-gradient AS, LG AS is defined as AVA ≤ 1.0 cm^2^ with mean gradient < 40 mmHg, which are discrepancies that lead to diagnostic difficulty and uncertainty over whether AS is truly severe and that AVR is indicated (Figure 1). Excluding measurement errors, most frequently, the low gradient is due to a low LV outflow state, with low LVEF < 50%, as the transvalvular gradient is a calculated from the squared function of flow, leading to a normal gradient despite severe AS. LF-LG AS with reduced LVEF is hence known as classical LF-LG AS. In contrast, paradoxical LF-LG AS has preserved LVEF, and may be caused by significant LV concentric remodeling, leading to diastolic dysfunction and reduction in stroke volume. A third category of LG AS is the normal-flow LG AS, which may be caused by pseudo-severe AS, reduced arterial compliance and systemic hypertension leading to reduced gradient and inherent inconsistencies in echocardiographic severity markers [15].

### 3.4. Low-Flow Low-Gradient AS with Reduced LVEF

Classical low-flow, low-gradient (LF-LG) AS occurs in patients with reduced LVEF (<50%) and low transvalvular flow (SVi ≤ 35 mL/m^2^), and accounts for 5–10% of cases. In these patients, the challenge lies in distinguishing true severe AS (TSAS) from pseudo-severe AS (PSAS), where the valve is only mildly or moderately stenotic but the valve leaflets appear immobile due to inadequate opening force rather than true anatomical stenosis, or the valve area appears severely stenotic due to inherent limitations in the valve area equation under low-flow conditions. The prevalence of pseudo-severe AS in LF-LG AS varies in the literature from 5 to 35%, depending on the diagnostic criteria [16].

Dobutamine challenge in dobutamine stress echocardiography (DSE) may increase cardiac output and differentiate LF-LG AS and reduced LVEF patients into TSAS, PSAS and patients with lack of contractile reserve [17]. This was first reported by deFilippi et al., who studied 24 patients with low-gradient severe AS (AVA ≤ 0.5, mean gradient ≤ 30 mmHg) and LV dysfunction [18]. In true-severe AS, dobutamine increases flow and gradient with minimal change in AVA, and this was identified in 39% of patients. In pseudo-severe AS, AVA increases to >1.0 cm^2^ with gradient remaining <40 mmHg, which was reported in 28% of patients in the original study [18]. Dobutamine challenge could also be performed during cardiac catheterization to similarly distinguish patients with TSAS from PSAS. In 32 patients with LF-LG AS, all patients with a mean transvalvular pressure gradient > 30 mmHg and a Gorlin AVA ≤ 1.2 cm^2^ at peak dobutamine infusion were found to have severe aortic stenosis at surgery [19]. Based on the ESC guidelines, low-dose dobutamine stress echocardiography is recommended (class 1) to distinguish between true severe and pseudo-severe aortic stenosis (increase in AVA to >1.0 cm^2^ with the increased flow) and identify patients with no contractile or flow reserve [4]. In patients with TSAS, with increase in stroke volume (increase ≥ 20%), unchanged aortic valve area (≤1 cm^2^) and increase in transvalvular gradient (mean pressure gradient ≥ 40 mmHg or peak aortic jet velocity ≥ 4 m/s), AVR is indicated as a class I recommendation [4].

The diagnostic accuracy of DSE for TSAS and PSAS may be complicated by the individual variation in the hemodynamic response to flow augmentation with dobutamine challenge. The ability of DSE to predict TSAS showed significant heterogeneity between various LVEF subgroups, with markedly different optimal discriminatory thresholds of a mean gradient 30 mmHg being the best cutoff in patients with LVEF < 35% and 40 mmHg in those with LVEF > 35% [20]. While DSE is safe and leads to an increase in stroke volume in patients with low-gradient AS regardless of LVEF, the association between DSE gradients and AS severity assessed by cardiac computed tomography (CCT) demonstrates important heterogeneity depending on LVEF, with the highest accuracy in patients with LVEF < 35% [20]. DSE may be less reliable in moderate-to-mild LV dysfunction or preserved EF, and hence is not conventionally performed in patients with preserved LVEF.

CCT and DSE provide complementary information, and CCT is recommended particularly in patients with low increase in flow with DSE, and moderate-to-mild LV dysfunction, or preserved LVEF. Calcium scoring provides objective assessment of stenosis severity independent of flow and hemodynamics. A previous study of patients with mild to severe AS and EF > 40% (n = 179) and EF ≤ 40% (n = 49) found a good correlation between AV calcification score and AVA, and a cut-off of 1651 arbitrary units (AU) derived from the LVEF > 40% cohort also applied in the LVEF ≤ 40% subgroup in all but three cases, who all had AU > 1200 [21]. Comparing DSE and CCT, Mogensen et al. reported in patients with LVEF < 50% that 23% of patients were labeled as having pseudosevere AS by DSE but severe AS by CCT, supporting the use of CCT in patients in the mild-to-moderate LV dysfunction range [20]. The 2025 ESC guidelines recommend CCT calcium scoring to determine severity in LF-LG AS with both reduced and preserved LVEF [4]. Thresholds for severe AS are sex-specific, being >3000 Agatston units (AU) in men and >1600 AU in women, or >2000 AU/m^2^ and >1300 AU/m^2^ when indexed to body surface area. Clavel et al. compared aortic valve calcification (AVC), AVC indexed (AVCi) and AVC density (AVCd), the latter being indexed to the cross-sectional area of the aortic annulus calculated from LVOT diameter measured by echocardiography in concordant and discordant AS [22]. They found that AVCd had highest area under the ROC curve in severe AS with concordant gradient and normal flow compared to AVC and AVCi, although it did not reach statistical significance. A recent study by the same group confirmed stronger correlations of sex-specific ACVd, with thresholds of 467 and 334 AU/cm^2^ for men and women, respectively, better identified severe AS and mortality than AVC or AVCd on echocardiogram [23]. Although interscan variability in AVC score was reported to be around 10% [24], validation studies found similar cut-off scores for sex-specific AVC that predicted AVR and death [25], with high reproducibility on repeat imaging [26]. The sex-specific AVC thresholds were also validated in the Asian population, although AVCd was more accurate than AVC in predicting severe AS in Asians [27]. AVC density also has prognostic value, and high AVCd may predict greater improvement in mortality post-TAVR in patients with classical LF-LG AS [28].

The study by deFilippi et al. identified a third group that had no contractile reserve in 33% of patients, and they showed no significant improvement in LV function and changes in hemodynamic indices with dobutamine [18]. The presence of contractile reserve (increase in stroke volume ≥ 20%) is considered a favorable prognostic marker for surgical AVR. In the earlier studies, patients with an absence of contractile reserve showed high operative mortality (odd ratio of up to 10 compared to patients with contractile reserve) with surgical AVR [29], and the presence of contractile reserve and AVR were the only factors significantly predicting long-term survival. However, the presence of contractile reserve did not predict LVEF recovery in patients surviving to AVR [30], nor long-term survival post-AVR, with similar improvement in NYHA functional classes. With transcatheter AVR (TAVR), good post-procedural outcomes were observed in a multicenter TOPAS-TAVI registry [31], with LVEF improvement, and the absence of contractile reserve on dobutamine stress echocardiogram did not predict clinical outcomes or LVEF changes over time. Sato et al. similarly identified that flow reserve on DSE was not significantly associated with survival after SAVR or TAVR, and AVR was associated with survival irrespective of flow reserve [32]. The lower procedural risk and expanding experience of TAVR offers a rationale for more liberal intervention, especially in symptomatic or high-risk patients even if they do not have classical flow reserve. Hence, the absence of contractile reserve should not be an exclusionary criterion to AVR, but rather a phenotype- and risk-based, individualized approach should guide decision making. Overall, in clinical practice, patients with classical LF-LG have poor prognosis, comparable with or worse than high-gradient AS [33]. Intervention should be considered in these patients with class IIa recommendation, particularly in patients with high calcium AV scoring on cardiac computed tomography (CCT) [4].

### 3.5. Paradoxical Low-Flow Low-Gradient AS with Preserved LVEF

Perhaps the most clinically significant advance in AS classification has been the recognition of paradoxical low-flow, low-gradient AS despite preserved LVEF, accounting for 10–25% of cases. This counterintuitive entity was systematically described by Hachicha et al. in 2007 and represents patients with severe AS (AVA ≤ 0.6 cm^2^/m^2^), preserved LVEF (≥50%), and low LV flow output defined as SVi ≤ 35 mL/m^2^ [12]. To identify true severe LFLG AS with preserved LVEF, accurate measurement of the AVA must be made, and error in left ventricular outflow tract (LVOT) diameter may lead to misclassification, particularly in the setting of upper septal hypertrophy [7,8]. For example, in a study of 108 patients with severe AS based on TTE, measurement of LVOT on transesophageal echocardiography (TEE) resulted in a decrease in proportion of patients with paradoxical LF-LG AS from 15% to 6%, due to reclassification to moderate AS [34]. Historically, the concept of ‘true severe AS’ was used primarily in classical LF-LG AS with reduced EF. With emerging evidence including the work of Hachicha and Pibarot, paradoxical LF-LG AS represents a distinct but equally high-risk phenotype where stenosis can be truly severe despite preserved LVEF. In cases with inconsistent aortic valve indices, invasive hemodynamic measurements may be made to guide the management of these cases, including measurement of SVi and Zva. In the literature, values of ZVa ≥ 5.0–5.5 mmHg/(mL/m^2^) there were worse clinical outcomes, hence studies have adopted cut-offs in this range [35,36,37]. To address the concern of inaccuracies in measurements on Doppler echocardiography, studies using invasive measurements were performed. The study by Mohty et al. on patients with severe AS and preserved LVEF assessed on cardiac catheterization found that patients with LF-LG AS, compared to those with normal flow high gradient, were older, with reduced systemic arterial compliance and vascular resistances and increased valvulo-arterial impedance (Zva) [38], confirming the findings of Hachicha et al. in 2007 [12]. Integration of multidetector computed tomography (MDCT)-derived measurements may be helpful in cases with discordant classifications. In a retrospective study of 307 cases with normal-flow, high-gradient calcific AS, MDCT planimetric AVA outperforms conventional AVA calculated from echocardiographic LVOT diameter and increased concordant classification of severe AS by 8% [39]. However, direct evidence for the use of MDCT-derived AVA in LF-LG AS remains lacking and further research is required. Based on ESC 2025 guidelines, paradoxical LF-LG AS patients should undergo an integrated assessment complemented by CCT AV calcium scoring to determine if there is severe AS [4].

Patients with paradoxical LF-LG AS were more likely female, with a lower transvalvular gradient, lower LVEF and higher level of LV global afterload reflected by a higher valvulo-arterial impedance compared to those with normal flow, and significantly, they also had a lower overall 3-year survival [12]. The increased global LV afterload and low output state evidenced by mean transvalvular flow rates were comparable to classical LF-LG AS. Greater increase in global LV afterload is associated with a combination of similar stenosis severity and lower systemic arterial compliance in paroxysmal compared to normal flow AS, with reduced myocardial contractility and more pronounced LV concentric remodeling, resulting in reduced stroke volume and cardiac output. A similar pattern of increased concentric remodeling and less eccentric hypertrophy was observed in up to 1/3 of mild to moderate AS, resulting in low flow [40]. Overall, in paradoxical LF-LG AS Hachicha et al. observed that increasing age, increasing valvulo-arterial impedance and medical management were associated with increased mortality [12].

LF-LG AS was also associated with increased 10-year mortality, with higher operative mortality for AVR but those undergoing AVR had better long-term survival than those managed conservatively [38]. Some earlier studies in the pre-TAVR era found that compared to patients with classical LF-LG AS, paradoxical LF-LG AS that is conservatively managed had lower mortality [41], and that only patients with high-gradient AS had statistically significant benefit with SAVR, and not patients with LF-LG AS [42]. In the prospective Simvastatin and Ezetimibe in Aortic Stenosis (SEAS) study, patients with low-gradient “severe” aortic stenosis (AVA < 1.0 cm^2^; mean pressure gradient ≤ 40 mm Hg) were compared with outcomes in patients with moderate stenosis (AVA 1.0 to 1.5 cm^2^; mean gradient 25 to 40 mm Hg). Aortic valve events, including death from cardiovascular causes, aortic valve replacement, and heart failure due to aortic stenosis, occurred in 48.5% of those with LG AS compared to 44.6% in those with moderate AS with no statistically significant difference [43]. It should be noted that this study was originally designed to include patients with mild to moderate asymptomatic AS with exclusion of severe AS and symptomatic patients, and only 51% of the LG group had SVi ≤ 35 mL/m^2^, which may not represent the typical LF-LG severe AS population. On the other hand, Clavel et al. compared patients with paradoxical LF-LG, HG and moderate AS, and found that SAVR was significantly associated with improved survival in HG AS and paradoxical LF-LG AS but not in moderate AS [44]. In the age of TAVR, intervention was generally associated with improved outcomes in patients with paradoxical LF-LG AS. Prakash et al. compared the outcomes of classical and paradoxical LF-LG AS, and found that in classical LFLG AS, TAVR significantly reduced mortality, but in paradoxical LFLG AS, a nonsignificant trend towards benefit was observed [41]. Another study reported improvements in overall mortality post-AVR for all patients with severe AS and normal LVEF, but the extent was the greatest in HG AS, followed by paradoxical LF-LG AS, and lowest in NF-LG AS, with a risk reduction of 84, 75, and 71%, respectively [45]. Hence, the benefit of AVR in paradoxical LF-LG AS may be lower than HG AS or classical LF-LG AS. A summary of the studies on AVR in patients with LF-LG AS is shown in Supplementary Table S1.

Outcomes post AVR tend to be poorer in LF-LG AS than HG AS. The PARTNER randomized controlled trial found that low flow, but not low LVEF (hence classical versus paradoxical LF-LG AS) or low gradient, is an independent predictor for early (30-day) and late mortality (2-year) after TAVR in high-risk patients with severe AS, although the 1-year mortality of paradoxical LF-LG AS was reduced from 66% to 35% after TAVR [46,47]. In a study of 312 patients with paradoxical LF-LG AS who underwent TAVR, 32% had died, had poor functional status or deterioration in function class, hence failing to derive benefit from TAVR, which was associated with low SVi [48]. Overall, a systematic review performed in 2015 involving 18 studies found that patients with paradoxical LF-LG AS have higher mortality than those with moderate AS (HR 1.68), NF-LG (HR 1.8) and HG (HR 1.67) AS. Although, it should be noted that AVR was associated with reduced mortality in patients with LF-LG (HR: 0.44), who were less likely to be referred to AVR compared to HG AS [49], hence patients with LF-LG AS may still benefit from AVR. Another meta-analysis in 2021 found that AVR improved survival in patients with classical or paradoxical LF-LG AS, and classical LF-LG AS had better outcomes post-AVR [50]. However, in conservatively managed patients, LVEF did not affect the survival or patients with LF-LG AS. Intervention is recommended in symptomatic patients with confirmed severe paradoxical LF-LG AS based on current guidelines; however, accurate diagnosis of true severe AS and patient selection is critical given the often worse outcomes post-AVR compared to other phenotypes.

### 3.6. Normal-Flow, Low-Gradient AS with Preserved LVEF

This phenotype is characterized by AVA ≤ 1.0 cm^2^, SVi > 35 mL/m^2^, mean gradient < 40 mmHg, and preserved LVEF. The discordance typically reflects truly moderate AS rather than severe disease, and the ESC 2025 guidelines classify this as likely moderate AS [4]. NF-LG AS is a heterogenous group that requires careful reassessment of AVA measurement and flow, symptomology and presence of hypertension. Additional investigation of AV calcification is helpful to distinguish true severe AS from pseudo-severe AS, and clinically relevant moderate AS (Figure 1). Therefore, results on the impact of AVR in this group of patients may differ. For example, studies have shown that even in the absence of low flow, symptomatic patients with this phenotype who undergo valve intervention have significantly lower mortality compared to conservative management [51]. In a study of NF-LG AS by Zusman et al., the 1-year mortality rate in the patients with NFLG who were treated conservatively in this study was 20%, compared to 10% for TAVR and 12% for SAVR [51]. Even after adjustment for age, LVEF and comorbidities, TAVR and SAVR was associated with lower mortality compared to conservative management, but no significant difference between TAVR and SAVR was found [51]. In a small observational study, 33% of normal-flow severe AS cases switch to low-flow on follow up, and this was predicted by higher initial valvuloarterial impedance, while 25% of low-flow patients switch to normal-flow associated with lower valvuloarterial impedance and higher systemic arterial compliance [52]. This highlights the importance of integrating clinical symptoms, comprehensive echocardiographic assessment including calcium scoring, and patient-specific factors on clinical follow up in decision-making.

### 3.7. Discordant High-Gradient AS

These patients show elevated gradients (mean ≥ 40 mmHg, Vmax ≥ 4.0 m/s) but AVA > 1.0 cm^2^. The elevated gradient may result from high-flow states (fever, anemia, hyperthyroidism, arteriovenous fistula, severe aortic regurgitation) or measurement error. Clinical assessment should exclude reversible high-flow conditions before classifying the stenosis as severe. If high flow is not reversible or explicable, these patients may indeed have severe stenosis despite the discordant AVA, and AVR should be considered.

## 4. Staging of AS Based on Cardiac Remodeling

Beyond flow phenotypes, recent studies focused on the effect of AS on the LV and other chambers, to stage AS based on the extent of cardiac damage in the setting of discordant Doppler-echocardiographic findings. In response to pressure overload in the LV, concentric remodeling and hypertrophy occurs, which chronically leads to increased myocyte volume and replacement fibrosis. Progressive fibrosis leads to diastolic dysfunction, loss of contractility and eventual systolic dysfunction, and impairment of atrial function, pulmonary hypertension, cardiogenic pulmonary congestion and right ventricular involvement [53,54].

Staging systems based on the extent of cardiac damage may have prognostic value. Based on the results of the PARTNER 2 trial, patients with severe AS undergoing AVR, the presence or absence of cardiac damage detected by echocardiography was significantly associated with one-year outcomes post-AVR [55]. Increasing cardiac damage from no extravalvular cardiac damage (Stage 0), left ventricular damage (Stage 1), left atrial or mitral valve damage (Stage 2), pulmonary vasculature or tricuspid valve damage (Stage 3), to right ventricular damage (Stage 4) showed increasing mortality with a hazard ratio (HR) of 1.46 per each increment in stage. This system has since been validated in different cohorts internationally [56,57,58,59,60], with development of several variations [61,62]. Okuno et al. subdivided Stage 3 to Stage 3a (≤moderate pulmonary hypertension) and Stage 3b (severe pulmonary hypertension), while Stage 4 was sub-divided into Stage 4a (low-flow without RV dysfunction), Stage 4b (RV dysfunction without low-flow), and Stage 4c (RV dysfunction with low-flow). Stepwise increase in 1-year mortality was also observed, but only Stage 3b, Stage 4b, and Stage 4c conferred a significantly increased risk of mortality compared to Stage 0–1. Another staging system with four stages based on mitral regurgitation (MR), left ventricle global longitudinal strain (LV-GLS) and right ventricular–arterial coupling (RVAc) on echocardiogram showed possibly superior predictability for mortality compared to the previous models [62].

However, conventional echocardiography has limited sensitivity in detecting subclinical myocardial damage from prolonged AS-related afterload and information from other techniques may be valuable [63]. Interestingly, concentric remodeling can also occur in mild to moderate AS, and is associated with low-flow AS with normal LVEF [40]. Novel indices of left ventricular remodeling, such as left ventricular stiffness, are found to be associated with LF AS, concentric remodeling and more severe diastolic dysfunction [64]. The reduction in global LV contractility index was associated with more severe AS despite preserved LVEF, and was independently associated with a higher composite outcome of aortic valve replacement, congestive cardiac failure admissions and all-cause mortality [65]. Whether these novel indices of cardiac remodeling may be an early marker for the development of severe AS with LFLG, and hence require increased vigilance for monitoring of indications for AVR, needs further investigation. Cardiac remodeling in severe AS may also be influenced by the existence of concomitant polyvalvular disease [66] and presence of other co-morbidities, an aspect that is poorly researched and understood. Post-intervention, cardiac damage and remodeling may also reverse, which may differ according to the phenotype of AS. In a large cohort study in Japan on 4523 patients, it was observed that 1 year post-TAVR, compared with HG-AS, classical LFLG AS showed smaller LV diameters, greater increase in LVEF, and comparable regression of LVMi, but not paradoxical LF-LG AS [67]. Further research should focus on integrating the use of cardiac damage-based staging with the phenotype-based classification, characterizing its utility in guiding the management of patients with discordant AS.

Advanced techniques including multi-chamber myocardial deformation analysis using speckle-tracking echocardiography (STE) can enhance current staging systems. LV global longitudinal strain (GLS) was found to be associated with all-cause mortality in severe AS, independent of the stage of cardiac damage, and had incremental prognostic value over clinical characteristics and stages of cardiac damage [68]. In a meta-analysis of 1067 asymptomatic patients with significant AS and normal LVEF, the best cutoff value identified was LVGLS of 14.7% (sensitivity, 60%; specificity, 70%) for prediction of mortality [69]. Tomaselli et al. proposed a novel staging system incorporating left ventricular, left atrial, and right ventricular strain parameters [70]. This multi-chamber approach improves risk stratification for patients with moderate-to-severe AS, potentially identifying those who would benefit from earlier intervention or require closer monitoring. Progressive cardiac damage across multiple chambers indicates more advanced disease and poorer prognosis, even in patients who might be classified as having moderate AS by traditional criteria.

## 5. Conclusions and Future Perspectives

With advancements in imaging acquisition and analysis techniques, classification of AS may be done with greater predictability for the need of AVR and with greater efficiency. Several AI-based models were described in the literature, for example one which could predict progression of AS to severe AS with accuracy of 83% using echocardiography [71]. Another fully automated assessment of AS on echocardiography closely matched trained echocardiographers’ measurements of aortic valve peak velocity, mean pressure, aortic valve area by continuity equation, stroke volume index, left ventricular outflow tract velocity-time, aortic valve velocity-time integral, and left ventricular outflow tract diameter, achieving 95.3% accuracy in correctly grading severe AS patients against the gold standard AVA by continuity equation [72]. Strom et al. evaluated an AI-based decision support to identify severe AS patients based on echocardiographic phenotypes, without relying on the LVOT measurements, which may be prone to measurement errors [73]. They found that the algorithm had excellent performance in identifying the phenotype associated with AVA < 1 cm^2^, and severe AS even in those with reduced LVEF. Importantly, the algorithm also identified 3.3% of individuals with guideline-defined moderate AS but with a similar clinical and TTE phenotype to those with severe AS, who had similar five-year mortality to those with known severe AS. These patients had low rates of AVR (6.6%) and may be a population with underrated AS severity. Since 2014, there was a significant increase in publications on artificial intelligence in aortic stenosis, particularly in diagnostics and risk prediction [74]. At present, the clinical adoption of AI algorithms in the diagnosis and prognostication of AS remains limited due to the lack of regulatory approvals, and more evidence for their validation is required.

The classification of AS has evolved to incorporate flow and LV function. The phenotype-based system highlights that low-flow, low-gradient AS represents a high-risk phenotype requiring comprehensive assessment, with multimodality imaging, particularly cardiac CT calcium scoring and dobutamine stress echocardiography, and advanced imaging techniques including speckle-tracking strain analysis, can detect subclinical cardiac damage and enhance risk stratification. In the future, the integration of phenotypic classification with advanced imaging, biomarkers, and AI-based tools may better identify the optimal timing for intervention in each patient, balancing the risks of premature valve replacement against the consequences of delayed treatment and irreversible cardiac remodeling.

## Figures and Tables

**Table 1:** Summary of the traditional grading criteria for AS severity based on hemodynamic parameters on echocardiography [11].

Parameter	Mild AS	Moderate AS	Severe AS
Peak velocity (m/s)	2.6–2.9	3.0–4.0	≥4.0
Mean gradient (mmHg)	<20	20–40	≥40
AVA (cm^2^)	>1.5	1.0–1.5	<1.0
Indexed AVA (cm^2^/m^2^)	>0.85	0.60–0.85	<0.60

**Table 2:** Phenotype classification of AS based on transvalvular gradient, flow and LVEF [4]. LF-LG = low-flow, low-gradient; NF-LG = normal-flow, low-gradient; SVi = stroke volume index.

Parameter	Concordant High-Gradient	LF-LG Reduced LVEF	LF-LG Preserved LVEF	NF-LG Preserved LVEF	Discordant High-Gradient
Mean gradient	≥40	<40	<40	<40	≥40
Peak velocity	≥4.0	-	-	-	≥4.0
AVA (cm^2^)	≤1.0	≤1.0	≤1.0	≤1.0	>1.0
SVi (mL/m^2^)	Any	≤35	≤35	>35	Any
LVEF (%)	Any	<50	≥50	≥50	Any

## Data Availability

No new data were created or analyzed in this study.

## Supplementary Materials

The following supporting information can be downloaded at: https://www.mdpi.com/article/10.3390/medicina62050819/s1, Table S1: Summary of studies on the impact of phenotype-based classification on outcomes post-surgical or transcatheter aortic valve replacement.
