# Supplementary material for: Categories of Aortic Stenosis: What’s New and the Clinical Implications
Source: Medicina (Kaunas). 2026 Apr 25;62(5):819. doi: 10.3390/medicina62050819 (PMC13208692; doi:10.3390/medicina62050819)
Supplement: Supplementary file 1 [file medicina-62-00819-s001.zip › medicina-4225760-supplementary.pdf]

Supplementary Table S1: Summary of studies on the impact of phenotype-based classification on outcomes post-surgical or transcatheter aortic valve replacement

Study, year	Study design	Population	Intervention	Follow up duration	Clinical outcomes	Results
Abramowitz et al. 2017	Retrospective observational study	770 patients with severe AS (357 NF, 413 LF)	TAVR	1 year	All-cause mortality	NF and LF had similar one-year mortality. NF and/or HG had lower one-year mortality than paradoxical LF-LG or classical LF-LG. Low mean gradient was an independent predictor of all-cause mortality.
Ahmed et al. 2017	Retrospective observational study	297 patients (129 NF-HG, 168 LF – 25 classical LF-LG and 143 paradoxical LF-LG)	TAVR	2.4 years	Symptoms on KCCQ, all-cause mortality, hospitalisation for heart failure	All groups had moderate improvements in quality of life at 1 month. No different in mortality at 2.4 years. Paradoxical LF-LG AS had higher risk of rehospitalization for heart failure.
Annabi et al. 2020 – TOPAS study	Prospective observational study	481 patients with LF-LG AS (72% classical LF-LG, 28% paradoxical LF-LG)	SAVR/TAVR; early AVR is ≤3 months	36 months	All-cause mortality	Survival benefit with early AVR in classical and paradoxical LF-LG AS, including pseudosevere AS. TAVR using femoral access had the best survival, followed by SAVR and then alternative-access TAVR.
Belin et al. 2024	Retrospective observational study	523 patients	TAVR (n = 303), SAVR (n = 220)	2011-2018	All-cause mortality	Patients with classical LF-LG and paradoxical LF-LG AS has higher prevalence of diastolic dysfunction, independent of AVR modality and increased risk of death.
Bellino et al. 2024	Retrospective observational study	392 patients with LF-LG AS	TAVR	1 year	All-cause mortality	No significant interaction of the classical and paradoxical LFLG-AS subgroups for all outcomes of interest.<br>RV dysfunction associated with increased mortality.
Besir et al. 2025	Retrospective observational study	633 patients with LF-LG AS	TAVR	30 days	Stroke volume index improvement by 20% or more, mortality, heart failure hospitalization	One-third of patients with LF-LG AS show an improvement of flow post-TAVR. Paradoxical LFLG AS, male sex, lower baseline SVI, and improvement in LVEF correlated with flow improvement. There was no difference in clinical outcomes between patients with and without flow improvement post-TAVR.

Besir et al. 2025	Retrospective observational study	1866 patients	TAVR	30 days	Mortality, heart failure hospitalization	No difference in heart failure hospitalization and mortality between patients with classical LF-LG, paradoxical LF-LG, NF-LG, HG AS
Brown et al. 2025	Retrospective observational study	575 patients with LF-LG AS	TAVR (n = 117), SAVR (n = 131)	30 days	Mortality, heart failure hospitalization, stroke and pacemaker insertion, paravalvular leak	TAVR was similar to SAVR for mortality and heart failure hospitalization. TAVR was associated with higher incidence of paravalvular leak, but not mortality, stroke or pacemaker.
Buckert et al. 2018	Prospective cohort study	30 patients with symptomatic AS (11 HF-HG, 6 LF-LG, 13 paradoxical LF-LG)	TAVR	3 months	Longitudinal strain and longitudinal velocity on CMR	Longitudinal velocity exhibited the highest predictive power for the identification of a low-flow state. HF-HG had recovery of longitudinal strain. LF-LG had reduction of longitudinal strain and velocity at baseline, which improved after TAVR. Paradoxical LF-LG had preserved longitudinal strain but reduced longitudinal velocity, with no improvement after TAVR.
Cardaioli et al. 2024	Retrospective observational study	574 patients (73% HG-AS, 15% pLFLG-AS, 11% cLFLG-AS)	TAVR	5 years minimum	Mortality	Classical LF-LG had the worst long-term survival, pLF-LG and HG had similar survival rates on unadjusted analysis but not on adjusted analysis.
Chong et al. 2024	Retrospective observational study	171 patients with paradoxical LF-LG AS	TAVR (n = 21), SAVR (n = 39)	5 years	Composite of cardiac mortality or AVR, all-cause mortality	AVR in the Doppler acceleration to ejection time ratio ≥0.35 group was associated with reduced cardiac and all-cause mortality, but not in patients with ratio <0.35
Clavel et al. 2012	Prospective cohort study	187 patients with paradoxical LF-LG AS, 187 patients with moderate AS, 187 patients with HG AS	SAVR	4.2 years	All-cause mortality, cardiovascular mortality	AVR was significantly associated with improved survival in HG AS and paradoxical LF-LG AS but not in moderate AS.
Dahan et al. 2025	Retrospective observational study	1189 patients with paradoxical LF-LG AS	AVR	5 years	All-cause mortality	Severe MR correlated with higher all-cause mortality. AVR significantly reduced mortality at every MR degree, with stronger impact with increasing MR degree.

Dahou et al. 2017	Prospective cohort study	32 patients with paradoxical LF-LG AS	TAVR (n = 9), SAVR (n = 23)	1 year	Global LV longitudinal strain, SVI	SVi increased, LVEF remain unchanged, flow normalised in 56% of patients at 1 year. SVi increased in patients with mild and moderate diastolic dysfunction but not patients with severe dysfunction. GLS increased at 1 year. Factors independently associated with SVi at 1 year post-AVR pre-operative SVi and presence of severe diastolic dysfunction.
Debry et al. 2016	Retrospective observational study	262 patients with severe symptomatic AS (31 pLF-LG, 172 HG, 59 cLF-LG)	TAVR	13.2 months	All-cause and cardiovascular mortality	Paradoxical LF-LG AS survival was similar to HG AS, classical LF-LG AS had higher all-cause and cardiovascular morality. 30-day mortality was significantly higher in paradoxical and classical LF-LG than HG AS.
Doldi et al. 2026	Retrospective observational study	2658 patients (211 HG, cLF-LG 206, pLF-LG 104)	TAVR	3 years	All-cause mortality	Atrial functional MR and primary MR was most common in pLF-LG but ventricular functional MR was most common in cLF-LG. MR severity affected 3-year mortality, with differences in patients with HG and pLF-LG but not cLF-LG AS. MR improvement was most commonly found in HG and least commonly found in pLF-LG patients.
Evertz et al. 2023	Prospective cohort study	92 patients (29 normal EF HG, 13 low EF HG, 25 low EF LG, 15 pLF-LG)	TAVR	600 days	Cardiovascular mortality	HG showed largest amount of AV calcification, compared to LG phenotypes. Myocardial fibrosis was most prevalent in low-output phenotypes (low EF LG > low EF HG > pLF-LG, normal EF HG). Cardiovascular mortality was greatest in low EF LG and pLF-LG AS. In patients with large myocardial fibrosis burden, those with large AV calcification have better outcome after TAVR.
Fiore et al. 2025	Retrospective observational study	408 patients with LF AS	TAVR (n = 245), SAVR (n = 57)	15 months	Composite of all-cause death and hospitalizations for heart failure	LF aetiology was not associated with outcomes. Severe MR was associated with primary endpoint, while AVR and MG ≥40mmHg were protective.
Fischer-Rasokat et al. 2019	Retrospective observational study	136 patients with HG or LF-LG	TAVR	1 year	All-cause mortality	Mortality in patients with LF-LG AS was twice that of HG AS patients.

		226 patients with HG or pLF-LG				
Fischer-Rasokat et al. 2021	Retrospective observational study	173 cLF-LG, 233 pLF-LG, 244 NF-LG, 1142 HG AS	TAVR	1 year	All-cause mortality	Patients with low AVC had lower MPG, with no differences between phenotypes. Symptom improvement at 30 days was less pronounced in classical LF-LG AS with low AVC. Survival were similar among patients with low and high AVC in all 3 LG groups.
Fraccaro et al. 2022 – OBSERVANT II dataset	Prospective cohort study	420 patients with classical LF-LG AS	TAVR (n = 389), SAVR (n = 401)	1 year	All-cause mortality, combined endpoint of all-cause mortality and heart failure hospitalization	Thirty-day mortality was 3.1%. One-year all-cause mortality was 17.4%, and the composite endpoint was 34.8%. 30-day and 1-year outcomes were similar between TAVR and SAVR.
Freitas-Ferraz et al. 2020	Retrospective observational study	318 patients with pLF-LG AS	TAVR	1 year	All-cause mortality, poor functional status, deterioration in function class	Primary endpoint was reached in 32% of patients, and low SVi was predictive.
Galian-Gay et al. 2022	Retrospective observational study	147 pLF-LG, 752 HG, 492 NF-LG	TAVR (n = 172), SAVR (n = 685)	59 months	All-cause mortality	Overall mortality was 27.7% with no differences among groups. Overall mortality reduction post-AVR was greatest in HG, followed by pLF-LG, and finally NF-LG AS, with a risk reduction of 84, 75, and 71%, respectively.
Guzzetti et al. 2020	Retrospective observational study	601 HG, 405 NF-LG, 246 LF-HG, 238 pLF-LG	AVR	2.42 years	All-cause mortality	LF/HG exhibited the highest mortality after AVR, both LF/LG and NF/LG patients had comparable outcome to NF/HG.
Hachicha et al. 2007	Retrospective observational study	512 patients (331 NF, 181 LF)	SAVR	3 years	All-cause mortality	LF patients had lower 3-year survival. Age, valvulo-arterial impedence and medical treatment were independently associated with increased mortality.
Haum et al. 2023	Prospective cohort study	230 patients, HG, pLF-LG, cLF-LG, NF-LG AS	TAVR	6 months	Daily step count	HG-AS and paradoxical LFLG-AS showed a significant improvement of daily steps, classical LFLG-AS showed no statistically relevant improvement, NFLG-AS showed a numerical decline in daily steps without statistical significance.

Herrmann et al. 2013 – PARTNER trial	RCT	971 patients, LF in 530, LF and low EF in 225, cLF-LG in 147	TAVR, SAVR vs medical treatment	2 years	All-cause mortality	Mortality was higher in patients with LF than NF, but both groups improved with TAVR in the inoperable cohort. In the high-risk cohort, there was no difference between TAVR and SAVR. In pLF-LG, TAVR reduced 1 year mortality from 66% to 35%.
Hundal et al. 2026	Retrospective observational study	1683 patients (929 HG, 344 pLF-LG, and 410 NF-LG)	TAVR	1 year	All-cause mortality	Mortality was higher among patients with pLF-LG AS (15.5%) compared with HG (10.8%). No significant difference in survival at 5 years.
Kataoka et al. 2018 – OCEAN-TAVI	Retrospective observational study	723 patients (97 pLF, 38 pLF-LG)	TAVR	8.8 months	All-cause mortality, cardiovascular mortality	LF and pLF-LG was associated with increased all-cause and cardiovascular mortality compared to NF. SVi was an independent predictor of cardiovascular mortality.
Lauten et al. 2014 – GARY	Retrospective observational study	359 low EF LG, 640 pLF-LG, 1864 HG AS	TAVR	1 year	All-cause mortality	Low EF LF had higher mortality than HG AS, while pLF-LG had similar mortality to HG AS.
Lopez-Marco et al. 2017	Retrospective observational study	198 patients (66 cLF-LG, 132 pLF-LG)	SAVR	3.7 years	All-cause mortality, NYHA I-II, improvement in ventricular function	In-hospital mortality was 3% in cLF-LG and 2.3% in pLF-LG AS. One- and five-year mortality rates were significantly greater in the cLF-LG AS group. 90% of the total survivors were in New York Heart Association class I-II, and 51% of the patients in the cLF-LG AS group had an improvement in their ventricular function.
Ludwig et al. 2020	Retrospective observational study	526 patients (290 cLF-LG, 236 pLF-LG)	TAVR	3 years	All-cause mortality	High AVC density was associated with lowest 1- and 3-year mortality in cLF-LG but not pLF-LG.
Mangner et al. 2018	Retrospective observational study	1600 patients (789 LF)	TAVR	3 years	All-cause mortality, cardiovascular mortality	LF had higher 30-day and 3-year mortality. pLF-LG, cLF-LG and LF-HG AS ahd higher all-cause mortality than NF-HG and NF-LG. Surviving patients showed a similar improvement in symptoms regardless of phenotype.
Mohty et al. 2013	Retrospective observational study	768 patients (13% LF-LG, 50% NF-	AVR	10 years	All-cause mortality	LFLG-AS was independently associated with reduced long-term survival. Patients with LFLG-AS undergoing AVR had better long-

		HG, 22% NF-LG, 15% LF-HG)				term survival than those managed conservatively (5-year survival rate: 63 ± 6% versus 38 ± 15%).
Mosleh et al. 2021	Retrospective observational study	73 pLF-LG, 217 HG AS	TAVR	1 year	Treatment futility, survival, change in functional status, quality of life, echocardiographic LV reverse remodeling	No differences in mortality, KCCQ-OS, NYHA-based futility outcome. Echocardiographic evidence of reverse remodeling of the left ventricle in pL-LG AS group was observed by improvements in global longitudinal strain.
Muratori et al. 2023	Retrospective observational study	1208 patients (976 NF-HG, 107 pLF-LG, 125 cLF-LG)	TAVR	5 years	All-cause mortality	Similar 30-day mortality but cLF-LG had lower 5-year survival rate compared to pLF-LG and NF-HG.
O'Sullivan et al. 2013	Retrospective observational study	385 patients with invasive measurements (208 HG, 85 pLF-LG, 61 low EF LG)	TAVR	1 year	Cardiac death, all-cause mortality, NYHA functional improvement	No difference in all-cause death in all groups at 30 days and 1 year. NYHA functional improvement occurred in all groups. Compared with HGAS, LEF-LG had a higher 1 year cardiac mortality
Okuno et al. 2021	Prospective cohort study	202 LF-LG AS	TAVR	1 year	All-cause mortality, futility (combined all-cause mortality or NYHA III/IV)	All-cause death at 1 year occurred in 6.6% of high-gradient AS patients, 10.9% of LFLG-AS patients with high likelihood of true severe AS, and in 7.2% of those with intermediate likelihood. Only the absence of aortic valve area ≤0.8 cm2 emerged as an independent predictor of treatment futility.
Osinalde et al. 2024	Retrospective observational study	205 patients (138 HG, 34 pLF-LG, 21 cLF-LG, 12 NF-LG)	68.3% AVR (51.7% TAVR)	1 year	Heart failure admission, AVR and death	Despite no differences in intervention rate, more patients with pLF-LG (32.4% vs. 15.9%; p = 0.049) died.
Prakash et al. 2025	Retrospective observational study	490 patients (207 cLF-LG, 283 pLF-LG)	67% TAVR	19 months	Mortality	TAVR was associated with lower mortality in cLF-LG (HR 0.37), but not significant in pLF-LG (HR 0.62). Among patients managed conservatively, those with pLFLG AS had lower mortality than cLFLG AS.
Puls et al. 2025	Prospective cohort study	250 patients (107 normal EF HG, 36 low EF HG, 52 cLF-LG, 38 pLF-LG)	TAVR	3-5 years	MLHFQ score, all-cause and cardiovascular mortality	TAVR lead to a significant decrease in MLHFQ score and NT-proBNP levels in all subtypes except for pLF-LG. pLF-LG patients had the poorest survival among all subtypes

						(HR 4.2), and was independently predictive for cardiovascular mortality.
Raikar et al. 2025	Retrospective observational study	1173 patients (very LG, LG, HG AS)	TAVR	1 year	NYHA symptom improvement, KCCQ-SS, rehospitalisation, mortality	TAVR improves symptoms and quality of life in LG, including those with VLGAS. A lower degree of symptom improvement was observed in VLGAS at 1 year, primarily driven by those with normal flow states. Differences in mortality and rehospitalization were observed only in classical low-flow states.
Ravn et al. 2026 – COMPARE-TAVI 1 trial	RCT	979 patients (NF-low EF, NF-normal EF, LF-low EF, LF-normal EF)	TAVR	1 year	Symptoms and improvement in 6-minute walk distance, SVi	LF-normal EF was most symptomatic and showed less improvement in 6-minute walk distance compared to NF-normal EF. Both LF groups showed increased SVi.
Reinthaler et al. 2014	Retrospective observational study	150 patients (30 pLF-LG, 21 c-LF-LG, 99 HG)	TAVR	1 year	All-cause mortality, cardiovascular mortality. 6 minute walk test.	pLF-LG is associated with less improvement in 6-minute walk test. pLF-LG and cLF-LG showed increased 1-year all-cause and cardiovascular mortality compared to HG AS.
Rodriguez-Gabella et al. 2018	Retrospective observational study	493 patients (396 HG, 97 pLF-LG)	TAVR	6 months	Futility (death or poor functional status NYHA III/IV)	No differences in mortality but pLF-LG were more frequently NYHA III/IV. TAVR futility was more frequent in pLF-LG (24% vs 14%), with higher rehospitlisation for cardiovascular cause.
Roslan et al. 2024	Retrospective observational study	1346 patients (486 HG, 154 moderate AS, 97 NF-LG, 62 pLF-LG, 59 cLF-LG, 26 reverse-area gradient mismatch	SAVR	1 year	All-cause mortality	In-hospital mortality was highest for cLF-LG (13.6%), followed by pLF-LG (11.3%), NF-LG (8.2%), HG (6.0%), moderate AS (5.2%) and reverse-area gradient mismatch (3.8%). For overall 1-year mortality, the highest was still cLF-LG (28.9%), followed by pLF-LG (24.0%), NF-LG (16.7%), HG (14.2%), moderate AS (13.2%) and reverse area-gradient mismatch (12.5%).
Rusinaru et al. 2021	Retrospective observational study	1397 patients (1271 HG, 126 pLF-LG)	AVR	5 years	All-cause mortality	AVR reduced mortality overall (HR 0.31), in HG AS (HR 0.18), and pLF-LG (HR 0.33).
Saito et al. 2021	Retrospective observational study	245 NF-HG, 48 pLF-LG	TAVR	518 days in NF-HG,	All-cause mortality, hospitalisation for valve-related	All-cause mortality, hopistlization for valve-related symptoms or worsening heart failure,

				400 days in pLF-LG	symptoms, worsening heart failure and NYHA III/IV	NYHA III/IV after TAVR was more frequent in pLF-LG patients than HG AS patients.
Salaun et al. 2020 – PARTNER 2	RCT and observational registry	3511 patients (2229 HG, 689 cLF-LG, 346 NF-LG)	TAVR, SAVR	2 years	Composite of death, heart failure or valve complication hospitalisation, stroke	Primary endpoint was higher in cLF-LG (38.8%) vs HG (31.8%) and NF-LG (32.1%), but similar to pLF-LG (33.6%). No significant difference between TAVR and SAVR within each flow-gradient group.
Schewel et al. 2016	Retrospective observational study	676 patients (HG, pLF-LG, cLF-LG)	TAVR	1 year	Mortality, improvement of functional capacity (NYHA), NTproBNP levels	CLFLG AS demonstrated a higher 30-day and 1-year mortality (NFHG/PLFLG/CLFLG 30-day: 5.9/9.6/18.3 %; 1-year: 11.4/22.4/38.2%). Nearly all survivors demonstrated an improvement of functional capacity (NYHA class) and a decrease of NTproBNP levels during the follow-up.
Schewel et al. 2020	Retrospective observational study	600 patients (296 NF-LG, 153 pLF-LG, 151 cLF-LG)	TAVR	5 years	Global afterload, SVI, overall survival, symptoms	pLF-LG and cLF-LG showed a significant reduction in global afterload and elevation of SVi. NF-LG was associated with an elevation of global afterload and a decrease of SVI ($p<0.05$). Overall survival was highest in NF-LG, followed by pLF-LG and cLF-LG. All subgroups experienced similar symptomatic improvement.
Simone et al. 2023	Retrospective observational study	304 patients (cLF-LG, pLF-LG, NF-LG, HG)	TAVR	1 month	KCCQ-12, all-cause mortality	All-cause mortality did not differ significantly among the groups at 1 month after surgery, and all groups experienced a significant improvement in quality-of-life scores after surgery.
Steffen et al. 2022	Retrospective observational study	1776 patients (956 HG, 447 cLF-LG, 373 pLF-LG)	TAVR	3 years	All-cause mortality, cardiovascular mortality VARC-3 composite endpoint, NYHA class	VARC-3 endpoints were similar, and NYHA class improved equally in all groups. Compared to HG, LF-LG patients had a higher 3-year all-cause mortality (cLF-LG HR 2.16 [1.77-2.64], pLF-LG HR 1.53 [1.22-193]), as well as cardiovascular mortality (cLF-LG, 2.88 [2.15-3.84], pLF-LG, 2.08 [1.50-2.87]).

Taniguchi et al. 2024 – CURRENT AS Registry 2	Prospective cohort study	3363 patients (285 low EF LG, 220 pLF-LG, 872 NF-LG, 1986 HG)	TAVR	3 years	All-cause mortality, cardiovascular death, NYHA III/IV	Overall survival at one and five years was highest in NFLG-AS. Freedom from cardiovascular death at one year was similar in NFLG-AS and PLFLG-AS, and higher in CLFLG-AS. NYHA class differed significantly among the subgroups, with the most common occurrence of NYHA Class ≥III being in CLFLG-AS, the functional class after TAVR was not significantly different among the three subgroups at 6 and 12 months.
Theut et al. 2017	Retrospective observational study	617 patients	TAVR	38.8 months	Mortality	HR for late mortality was 0.98, 1.11, and 1.90 for the low-, intermediate-, and high-risk TAVR groups, respectively, as compared to their matched controls and 1.04, 1.45, and 1.52 for the high gradient, pLF-LG, and cLF-LG groups, respectively, as compared to their controls.
Wagener et al. 2023 - SwissTAVI	Prospective cohort study	8914 patients	TAVR	5 years	All-cause mortality	Lowest 1-year all-cause mortality in HG (8.8%), pLF-LG (11.5%), cLF-LG (19.8%). Cardiovascular death shows similar trend. At 5 years, the all-cause death rate was 44.4% in HG, 52.1% in P-LFLG, and 62.8% in C-LFLG aortic stenosis.
Wani et al. 2022	Retrospective observational study	204 patients (114 HG, 31 NF-HG low EF, 32 cLF-LG, 27 pLF-LG)	TAVR	1 year	Heart failure hospitlisation, cardiac mortality, all cause mortality	Patients with low-flow AS had similar improvements in LVEF and aortic valve mean gradient as normal-flow AS. GLS was significantly improved in patients with normal-flow compared to low-flow AS. Across all types of AS, improvement in GLS was associated with a survival benefit, with GLS recovery in alive patients.
Yokoyama et al. 2024	Retrospective observational study	4523 patients (3697 HG, 507 cLF-LG, 319 pLF-LG)	TAVR	1 years	LV dimensions, LVEF, LV mass index, SVi	During 1 year post-TAVI, compared with HG AS, cLFLG AS achieves smaller LV diameters, greater increase in LVEF, and comparable regression of LVMi, whereas pLFLG AS does not.

Zbronski et al. 2019	Retrospective observational study	231 patients (42 pLF-LG, 40 cLF-LG, 149 HG)	TAVR	1 year	All-cause mortality	Overall, 46 (19.9%) patients died within 12 months after TAVR. The 30-day and 1-year survival was comparable between AS subtypes.

AS = aortic stenosis; cLF-LG = classical low flow low gradient AS; EF = ejection fraction; GLS = global longitudinal strain; HG = high gradient; KCCQ = Kasas City Cardiomyopathy Questionnaire; MG = mean gradient; NYHA = New York Heart Association; pLF-LG = paradoxical low flow low gradient AS; SAVR = surgical aortic valve replacement; SVi = stroke volume index; TAVR = transcatheter aortic valve replacement; Valve Academic Research Consortium-3 = VARC-3
